# Application of the RDoC Framework to Predict Alcohol Use and Suicidal Thoughts and Behaviors among Early Adolescents in the Adolescent Brain and Cognitive Development (ABCD) Study

**DOI:** 10.3390/brainsci12070935

**Published:** 2022-07-17

**Authors:** Laika D. Aguinaldo, Clarisa Coronado, Diego A. Gomes, Kelly E. Courtney, Joanna Jacobus

**Affiliations:** Department of Psychiatry, University of California, La Jolla, CA 92093, USA; laaguinaldo@health.ucsd.edu (L.D.A.); clari.coronado@gmail.com (C.C.); gomesadiego@gmail.com (D.A.G.); kecourtney@health.ucsd.edu (K.E.C.)

**Keywords:** RDoC, suicide prevention, suicide intervention, pre-teen youth, alcohol use

## Abstract

Alcohol use confers risk for suicidal thoughts and behaviors (ideation, attempt) in early adolescents. The Research Domain Criteria provides a framework for examination of multidimensional and modifiable risk factors. We examined distinct latent profiles based on patterns of positive valence (reward responsivity) and cognitive systems (neurocognition) from the ABCD Study (age 9–10, *N* = 10,414) at baseline enrollment. Longitudinal associations were determined between baseline positive valence and cognitive profiles and group classification (alcohol use, suicidal thoughts and behaviors, or their co-occurrence) two-years after initial assessment (ages 11–12). Three unique profiles of positive valence, cognition, alcohol use, and suicidal thoughts and behaviors were identified. Two baseline profiles predicted alcohol use and suicidal thoughts and behaviors, two-years after initial assessment. Low positive valence with high cognition (but low impulsivity) predicted alcohol use (OR = 1.414, *p* < 0.001), while high positive valence with low cognition (but high impulsivity) predicted suicidal thoughts and behaviors (OR = 1.25, *p* = 0.038), compared to average positive valence and cognition. Unique profiles of positive valence and cognitive systems among 9–12-year-olds may be predictive of alcohol use and suicidal thoughts and behaviors over a two-year period. Findings underscore the potential for trajectory research on positive valence and cognitive profiles to enhance prevention for early-adolescents.

## 1. Introduction

Alcohol use is linked to suicidal thoughts and behaviors during early adolescence (prepubescence) [1]. In our prior research, early adolescents who reported lifetime alcohol use (i.e., ≥a sip) showed a nearly two-fold increase in their odds of lifetime suicidal ideation and attempts, compared to early adolescents with no previous alcohol use [1]. Given that the alcohol use (ALC)–suicidal thoughts and behaviors (STB) association can be detected in early adolescence (as young as age 9), it is important to determine the mechanisms that may increase vulnerability to the ALC-STB association, given that the comorbidity is particularly deadly [2]. However, the extant literature on the ALC-STB association examines the same set of risk factors (e.g., internalizing, and externalizing symptoms, STB history, and demographic and culture/environment characteristics) studied in isolation often using cross-sectional designs with older adolescents, adult populations, and/or small sample sizes [3,4,5,6,7,8]. There is a need for research on the ALC-STB association that characterizes novel risk factors, examines the relationships that exist between them, and utilizes prospective designs with populations underrepresented in the comorbid ALC and STB literature (e.g., early-adolescents).

The National Institute of Mental Health has developed a research framework for investigating mental disorders, the Research Domain Criteria (RDoC). The goal of RDoC is to overcome one-dimensional limitations, and promote novel approaches that will lead to better diagnosis, prevention, and intervention. RDoC is a conceptual research framework that prioritizes neurobiological and transdiagnostic risk factors that are present across mental illness (e.g., STBs) [8,9,10], with a focus on risk factor integration across human functioning domains. The framework currently includes six major functional domains (arousal/regulatory, positive valence, negative valence, sensorimotor, cognitive, and social processes). Different aspects of each domain are represented by three to six psychological/biological dimensions, or constructs, which are studied along the full range of functioning from normal to abnormal. Both behavioral and biological aspects of functioning change and mature throughout pre-to post pubescence; thus, research during these critical development periods is important. The RDoC framework encourages researchers to measure and integrate many classes of variables (units of analysis, e.g., behavioral, physiological, and self-report data) in order to seek a comprehensive understanding of the construct(s) under study. Advancing the ALC-STB association literature by using this framework, coupled with advanced statistical methods, allows for the exploration of brain–behavior relationships using complex computational approaches to explore increasing risk for STBs [8,11].

Associations between the RDoC cognitive and positive valence system domains are understudied. Positive valence systems (PVS) are responsible for responses to positive motivational situations or contexts, such as reward-seeking, consummatory behavior, and reward/habit learning. A recent review by Glenn et al. (2018) used RDoC to examine relationships between cognitive and positive valence risk factors and STBs in adult samples [8]. Poorer working memory increased the risk for STBs, while findings for the association between cognitive control (e.g., impulsiveness, problem-solving (approach avoidance, personal control)) and STBs were mixed [8]. In the domain of positive valance, adolescents who self-reported STBs demonstrated blunted responses to reward responsiveness [7,12,13,14,15,16,17,18]. In a recent study that used an RDoC approach to test blunted positive valence in 57 adult men and women heavy binge drinkers compared to healthy controls during functional magnetic resonance imaging, binge alcohol drinkers showed abnormally blunted activity in PVS regions, such as the striatum, compared with healthy controls [19]. PVS brain regions are well established in the adult literature (e.g., striatum, dopamine, and opioid system) [19,20,21]. However, research is needed to further examine these understudied neuroscience-based risk factors using statistical models that consider complex relationships among several variables simultaneously and in pre–post pubescent samples [7,8,12,13,14,22,23].

Latent Profile Analysis (LPA) is a statistical approach that considers complex relationships among several predictors simultaneously. LPA has been used in adult populations to identify profiles (i.e., subgroups) of people who engage in STBs. Prior research has focused on patterns among self-reports of thoughts of death and suicide and healthy lifestyle patterns [24,25,26,27,28,29,30,31]. To our knowledge, only one study exists examining suicidality using the RDoC framework and LPA [10]. Podlogar and colleagues (2018) used LPA to assess overlapping and distinct features of depression and anxiety in relation to suicide risk among 616 adult outpatients [10]. Results demonstrated that those identified as showing a higher suicide risk profile reported significantly higher levels of negative affect and anxious distress and reported significantly lower levels of positive affect than other classes [10]. To date, the application of LPA models to studies of child and adolescent STBs within the RDoC framework is an understudied area of research.

Early life (prepubescent) ALC, STBs, reward responsivity, and cognitive control are critical risk factors that share similar characteristics and predict addictive and suicidal behaviors [1,3,7,8,12,13,14,15,16,17,18,22,23]. Driven by prior research, theory, and parsimony, a priori construct selection drove our hypothesis and aims [1,7,8,12,13,14,15,16,17,18,22,23,32]. The primary aims of this study were to examine latent profiles of (1) positive valence (PVS) and cognitive (CS) systems, ALC, and STBs among 9- to 10-year-olds in the ABCD baseline cohort and (2) examine if PVS, CS, ALC, and STB profiles prospectively predict ALC, STBs, and their co-occurrence two years after initial assessment. We hypothesized that profiles of high PVS and low CS would predict ALC, STBs, and their co-occurrence and that these patterns in profiles could predict future ALC, STBs, and their co-occurrence.

## 2. Materials and Methods

### 2.1. Protocol

ABCD is a 10-year longitudinal study conducted across 21 sites in the United States that recruited 11,878 participants and is funded by the National Institutes of Health [33,34]. The Institutional Review Board at the University of California, San Diego, approved the study, and each study site has a detailed protocol to address reports of STBs. A detailed account of the recruitment strategy has been previously published [35,36,37]. ABCD primarily utilized a probability sample recruited through schools, with school selection based on sex, race and ethnicity, socioeconomic status, and urbanicity.

Participants and their parents or guardians attended study session(s) at their local research site to complete the baseline and follow-up visits. Parents/guardians provided written consent, with each early adolescent providing written assent. Participants and their parent or guardian were in separate private rooms during study participation to maintain confidentiality. The baseline and year two measures included self- and parent/guardian-report questionnaires, neurocognitive testing, biological samples, and an MRI scan [38,39,40]. Study assessments were completed over an 8-h research session (or two 4-h sessions), and parents and youths were compensated financially. The cross-sectional and longitudinal analyses presented herein drew from self- and parent-report questionnaires and neurocognitive assessments from the baseline and year two follow-up data of the ABCD data (Release 4.0).

### 2.2. Measures

#### 2.2.1. Positive Valence Systems (PVS; Trait-Based Measures)

Sensitivity to Reward and Punishment: The 24 item BIS/BAS survey was administered to all youth to assess for avoidance (BIS) and approach (BAS) sensitivities reflective of motivational traits [41,42,43,44]. The cross-sectional LPA included the BIS summary and BAS reward responsiveness subscale scores. Scoring is based on youth self-reports per subscale (BIS, BAS reward responsiveness), such that high values on BIS correspond to high avoidance sensitivities, low values on BIS correspond to low avoidance sensitivities, and high values on BAS reward responsiveness correspond to high approach sensitivities to reward responsiveness, and low values on BAS reward responsiveness correspond to low approach sensitivities to reward responsiveness.

#### 2.2.2. NIH Neurocognitive Toolbox (Cognitive Systems; CS)

Inhibitory Control and Attention-Flanker (uncorrected standard score): The NIH Toolbox Flanker Inhibition Control and Attention Task, a variant of the Eriksen Flanker task, was used to measure attention and the ability to inhibit automatic responses that interfere with achieving goals [45]. Participants were presented with five arrows on the iPad screen with four flanking stimuli (two on the outer left and two on the outer right) all facing the same way, either left or right. The middle arrow either faced the same way (congruent trial) or a different way (incongruent trial) as the flanking stimuli. Participants pressed a button to indicate whether the middle stimuli faced left or right. The word MIDDLE also appeared on the screen for all participants. For participants ages 8 to 11 years, an audio recording stated “MIDDLE” to remind participants where to focus. Scoring is based on speed and accuracy (e.g., higher normative scores indicate better ability to attend to relevant stimuli and ignore irrelevant stimuli), with high values corresponding to higher cognitive control and attention, and low values corresponding to lower cognitive control and attention.

Processing Speed and Information Processing (uncorrected standard score): The NIH Toolbox Pattern Comparison Processing Speed Test^®^ [46] was used as the measure of rapid visual processing. Participants were shown two pictures and asked to determine whether the pictures were the same or not. High scores can be interpreted as a better and faster processing speed and low scores can be interpreted as a slower processing speed.

Episodic Learning and Memory-Picture Sequence Memory Task (uncorrected standard score; PSMT): The PSMT is modeled from memory tests asking children to imitate a sequence of actions using props and measures episodic memory for a sequence of pictured events [47,48]. Participants were presented with a series of pictures, not in any intrinsic order, depicting activities or events that could occur in a particular setting (i.e., going to the fair) [47,49]. After the last item was presented, the pictures were randomly displayed in the center of the screen, and the participants were asked to place the pictures in the same order they were presented. There were three different sets of test items for this measure: (1) Play in the Park (Form A), (2) Go to the Fair (Form B), and (3) Work on the Farm (Form C); each form consists of different test items that yield equivalent scores and can be used in a repeated-measures research design to minimize practice effects [47,48]. Form A (Play in the Park) was administered to all participants at baseline and Form B (Go to the Fair) was administered to all participants at the two-year follow-up. Scoring is based on the total number of adjacent pairs of pictures placed correctly across two learning trials (high values are interpreted as a better working memory, while low values are interpreted as a poorer episodic memory).

Verbal Learning. Rey Auditory Verbal Learning Test (short and long delay total correct raw score): The Rey Auditory Verbal Learning Test (RAVLT) was used as a measure of learning and memory across the lifespan that is sensitive to various influences, including psychopathology across development [50,51,52]. A customized automated version of the RAVLT, created with Pearson’s Q-interactive platform, was used [53]. Participants listened to and recalled a list of 15 unrelated words over five learning trials. Following the fifth initially learned list, participants are asked to listen and recall a distractor list, then they are asked to recall the initially learned list. To assess longer-term retention, recall is reassessed following a 30-min delay during which other non-verbal tasks from the neurocognitive battery were administered. Alternate forms were used at baseline and two-year follow-up to facilitate longitudinal testing [38]. Q-interactive automatically calculated the number of correctly recalled words for each trial and the number of perseverations and intrusions. A higher uncorrected raw score represents better verbal learning and recall performance, while lower uncorrected raw score represents poorer verbal learning and recall performance.

Impulsivity: The shortened 20-item youth version of the Urgency, Premeditation, Perseverance, Sensation Seeking, and Positive Urgency (UPPS-P) [54,55] scale was administered to youth at baseline and two-year follow-up. Recent research has demonstrated no significant differences in psychometric performance of this measure across gender, race/ethnicity, and socioeconomic status amongst youths [55]. High scores on each subscale can be interpreted as higher impulsivity, and low scores on subscales can be interpreted as lower impulsivity.

#### 2.2.3. Measures of Alcohol Use, Suicidal Thoughts and Behaviors, and Demographic Covariates

Lifetime Low-Level Alcohol Use (ALC): Youth completed the iSay Sip Inventory to characterize participants’ endorsement of low-level alcohol use (any alcohol drink and/or sip) [52].

Suicidal Thoughts and Behaviors (STBs): Youths’ reports of lifetime SI and SA were gathered from a computerized version of the Kiddie-Schedule for Affective Disorders and Schizophrenia (K-SADS) [56]. Participants at baseline and year two follow-up of the ABCD cohort were separated into three groups: (1) lifetime low-level alcohol use and no lifetime STBs were categorized as ALC, (2) no lifetime low-level alcohol use and lifetime STBs were categorized as STBs, and (3) the co-occurrence of lifetime low-level alcohol use and lifetime STBs were categorized as ALC + STBs.

Demographic Covariates: Demographic covariates were chosen based on prior evidence of associations with the ALC and STB variables [53,57,58,59,60,61,62,63,64,65,66] (Table 1). The parent-reported demographic variables of age, sex (assigned at birth), parental education, and ethno-racial identity, are items extracted from the PhenX toolkit [62].

### 2.3. Statistical Methods

Missing data, demographics, and covariates: ABCD data harmonization, multiple imputation, and data analyses were conducted in R (Version 1.2.5033, Auckland, New Zealand). Descriptive statistics were used to summarize baseline characteristics of the total sample (*n* = 10,414) and by ALC/STBs group (ALC, STBs, and ALC + STBs) prior to multiple imputation. Between-group differences among the different latent profiles (Profile 1: average PVS and CS vs. Profile 2: high PVS and low CS (high impulsivity), Profile 1: average PVS and CS vs. Profile 3: low PVS and high CS) were assessed using t-tests for PVS, CS, and age (treated as continuous variables), and chi-square tests for categorical variables (sex, parental education, and ethno-racial identity), with adjusted *p*-values due presented due to Bonferroni corrections from multiple comparisons; Table 1). Demographic covariates were included in the longitudinal analyses and included age, sex (biological), ethno-racial identity, and parent education [64,65,66,67,68,69,70,71,72].

Multiple imputation with chained equations using the mice package in R were used to obtain more complete data sets and to better protect against bias due to data missing at random mechanisms [72]. This process produced a total of 25 new data sets with the observed and imputed scores [73].

Cross-Sectional Baseline Latent Profile Analysis. To test for the existence of discrete profiles based on PVS, CS, and ALC/STBs patterns, we conducted an LPA at baseline enrollment (Table 2) using an imputed data set that was the most similar to the means and standard deviations in the total sample [74]. LPA is a model-based data reduction method for identifying latent profiles (i.e., subgroups) within a population based on response patterns among a set of variables (PVS, CS, and ALC/STBs) [75]. We employed LPA using all continuous positive valence variables (i.e., reward sensitivity), all continuous neurocognitive variables (i.e., language/verbal intellect, inhibitory control and attention, processing speed/information processing, episodic memory, reading ability/language/academic achievement, verbal learning, and impulsivity), and lifetime ALC/STBs variables (dichotomous).

In LPA, fit indices are compared across models to determine the optimal number of profiles, firstly evaluating a two-profile model fit and incrementally adding latent profiles up to a five-profile model until the best profile solution is found. The final model selection uses several fit indices, including *information criteria*, *likelihood ratios*, and *entropy*. For the information criteria, we used the Akaike Information Criterion (AIC) [75], and the Bayesian Information Criterion (BIC) [76], in which lower values in information criterion statistics indicate a better fit. We relied on the Lo-Mendell-Rubin adjusted likelihood ratio test (LRT) [77] of statistically significant difference (*p* < 0.05) to suggest the number of profiles that are the best fit. Lastly, we relied on a standardized measure of entropy which is an index of model-based classification accuracy. Entropy indicates how accurately the model defines the participant’s classification to a latent profile. Relying on the standardized entropy values range from 0 to 1, higher values indicate more precise assignment of participants to latent profiles, and better separation of identified profiles (e.g., entropy value close to 1 is ideal) [78,79].

Longitudinal Multinomial Logistic Regression Analysis. We used multinomial logistic regression models to test PVS, CS, and ALC/STBs baseline profiles as predictors of ALC, STBs, and ALC + STBs at year two follow-up. Specifically, our multinomial logistic regression model tested a nominal variable for profile class as the predictor of interest, with the reference group being the average PVS and CS group. Next, this nominal variable for each LPA profile was examined as a potential predictor of four of the outcome groups (NO ALC/STBs, ALC, STBs, and ALC + STBs; nominal outcomes) at year two follow-up. The longitudinal multinomial model controlled for demographic covariates of sex, ethno-racial identity, and study site.

## 3. Results

### 3.1. Descriptive Analysis

A total of 10,414 participants (average age 9.9 years, 52.2% male, 52% non-Hispanic White) who completed baseline enrollment and year two follow-up were included in the analyses (Table 1).

### 3.2. Cross-Sectional, Latent Profile Analysis

**Figure 1:**
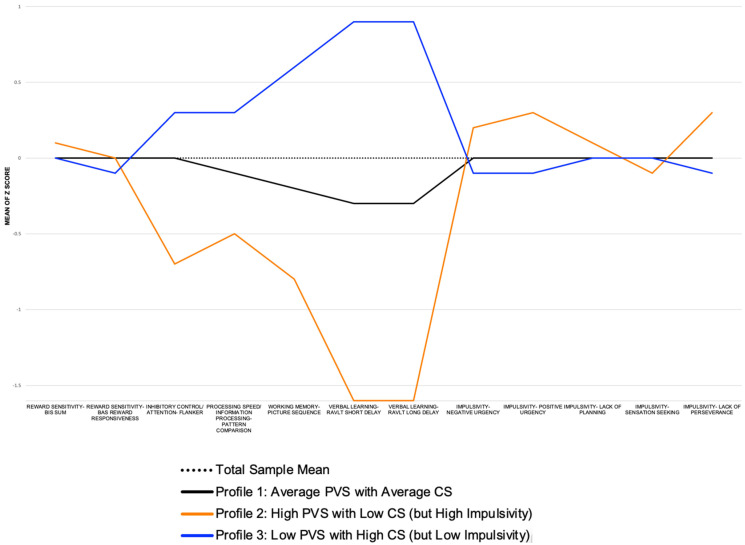
Positive valence and cognitive system domains (X Axis) and mean score (Y Axis) by profile at baseline enrollment in the Adolescent Brain and Cognitive Development study (*N* = 10,414).

Our data best supported a three-profile model based on recommended decision criteria for evaluating latent patterns (Table 2) [74]. Specifically, the model fit indices for the three-profile model indicated lower AIC and BIC values than the four-profile solution, a significant Vuong Lo Mendell Rubin adjusted *p*-value (*p* = 0.036), and an entropy value of 0.77). Each of the three profiles were adequately populated (profile 1, *n* = 4940; profile 2, *n* = 1470; profile 3, *n* = 4004; Table 1), which was not true for the other profile solutions. Furthermore, the three-profile model yielded conceptually meaningful configurations of participant profiles: (1) average PVS and CS, (2) high PVS with low CS (high impulsivity), and low PVS with high CS. The three profiles were statistically different (*p* = 0.036) in PVS, CS, ALC, and STBs, but not co-occurring ALC + STBs (Table 1, Figure 1).

### 3.3. Prospective, Multinomial Logistic Regression Analyses

A multinomial logistic regression analysis identified whether baseline PVS, CS, ALC, STBs, profiles significantly predicted ALC, STBs, and ALC + STBs two years after initial assessment. All three of the PVS, CS, and ALC STB profiles from the cross-sectional LPA were included as a nominal predictor (e.g., Profile 1: average PVS and CS = REFERENCE GROUP, compared to Profile 2: high PVS with low CS (but high impulsivity), and Profile 3: low PVS with high CS (but low impulsivity) in the multinomial logistic regression predicting classification to NO ALC/STBs (reference group), ALC, STBs, and ALC + STBs (Table 3). Two of the baseline PVS, CS, ALC, and STB profiles predicted ALC and STBs, assessed two years later. The low PVS with high CS (but low impulsivity predicted ALC (OR = 1.41, 95% CI 1.17–1.71, *p* ≤ 0.001), while high PVS with low CS (high impulsivity) predicted STBs (OR = 1.25, *p* = 1.01–1.56). The multinomial logistic regression controlled for baseline demographics of age (*p* ≤ 0.001 ALC, *p* = 0.024 STB, *p* = 0.322 ALC + STBs), sex (*p* = 0.001 ALC, *p* ≤ 0.001 STB, *p* = 0.002 ALC + STBs), ethno-racial identity (*p* ≤ 0.001 ALC, *p* = 0.05 STBs, *p* = 0.07 ALC + STBs), and parental education (*p* ≤ 0.001 ALC, STBs, and ALC + STBs).

## 4. Discussion

This study demonstrated that the combination of the RDoC framework and data-driven advanced statistical modeling provides a novel and promising approach to research on the ALC-STBs association in early adolescence. We found two conceptually meaningful participant profiles of PVS and CS among youth ages 9–10 at the baseline time point. We found evidence of clinical meaning as the unique profile of low PVS with high CS (but low impulsivity) was prospectively associated with ALC at two-year follow-up; while the profile of high PVS with low CS (but high impulsivity) prospectively predicted STBs independently at two-year follow-up, implying that early adolescents who endorse STBs have high PVS and low CS (but high impulsivity) at ages 9–10 and are at increased risk for suicide at ages 11–12. This finding has not been well researched in the early adolescent literature, and while the simultaneous modeling of the ALC-STB association using PVS and CS constructs is sparse, research on adult populations showing the high PVS-STB link has been replicated [4] while the low CS-STB findings have been less consistent [4,6].

To our knowledge, this is the first study to examine unique profiles of PVS, CS, ALC, and STB patterns simultaneously using data-driven, advanced statistical modeling in children as young as 9–10-years. Notably, the most common (*n* = 4940, ~47%) PV/CS/ALC/STB profile among children in the ABCD baseline cohort can be described as the average PVS and CS group, followed by the low PVS and high CS (but low impulsivity; *n* = 4004, ~38%), and the least common profile was the high PVS with low CS (but high impulsivity; *n* = 1470, ~14%). Few studies have explored PVS and CS profiles and their ability to predict alcohol use, suicidal thoughts and behaviors, and their co-occurrence at a later time point, thus it is difficult to make comparisons with other research. However, the characterization of unique PVS and CS profiles might facilitate a deeper understanding of the ALC-STB association and inform future research and targets for intervention.

These findings of neurocognitive and behavioral profiles have notable clinical relevance in both ALC and STB prevention and intervention. First, these findings may inform future research on the screening and detection of ALC, STBs, their co-occurrence, and risk factors, in youth as young as 9 years of age. A tiered approach to screening with validated (population- and setting-specific) instruments that identify increased ALC and STBs risk may benefit from considering combined neurocognitive (language/vocabulary comprehension, cognitive control, episodic memory, and impulsivity), and positive valence (reward sensitivity) assessment [80,81]. Second, patterns in positive valence and cognitive system deficits may influence treatment outcomes, thereby making patients less amenable or responsive to psychotherapeutic approaches that have demonstrated effectiveness at treating ALC, STBs, or their co-occurrence. Thus, thoughtful consideration in tailoring interventions to address the positive valence and neurocognitive heterogeneity is important. It may be that patients who present with deficits in these domains, could benefit from adjunctive treatments directed at ameliorating deficits in positive valence and cognitive systems, such as cognitive training. Third, given the co-occurrence of ALC and STBs in youth as young as 9 years, personalized treatments to meet this high-risk group are needed. To date, there is currently no standard approach to treating co-occurring ALC and STBs, but motivational enhancement therapy or cognitive behavioral therapy approaches have demonstrated efficacy [82,83]. Unfortunately, ALC and STBs are often treated in two separate systems of care, and usually with older adolescents, highlighting an important and unmet need [82,83]. However, one intervention to highlight is a two-site randomized control trial that recruited 95 participants aged 13–21, with depression and substance use, led by John Curry, PhD, MD, and Yifrah Kaminer, MD, MBA at the University of Connecticut (J. Curry, personal communication, 14 June 2022) [83]. The trial includes an adaptive method, such that initial substance use treatment (CBT) is supplemented with one of two depression treatments (CBT-for-depression [CBT-D], or treatment-as-usual [TAU] only when needed. Study findings included that 35 of the 95 adolescents had positive depression response to CBT for substance use within one month; and that for the remaining adolescents, there were no differences in depression or substance use outcomes between CBT-D and TAU at the end of treatment. All groups showed reduced depression and reduced substance use. Although this trial excluded youth who were at high risk for suicide (past-month SA or current SI with intent or plan), the investigators did include measures that assess STBs, that they plan to examine in subsequent work. This research is both timely and novel given the increase in STBs, and early adolescent youth as young as age 9 incur additional risk when ALC co-occurs. Interventions that can treat the co-occurrence of ALC and STBs in youth as young as age 9 is an important and understudied area of research.

While this study’s findings are critical additions to the literature, limitations exist. As these analyses include two time points (baseline study enrollment, year two follow-up), directionality and causal inferences cannot be conclusively derived. Patterns of PVS and CS may be due to other mechanisms (e.g., family history of STBs, psychiatric diagnosis). In follow-up prospective ABCD analyses, we will extrapolate the directionality of the relationship between decrements in PVS, CS, ALC, and STBs longitudinally while examining potential confounds within-group differences (children and adolescents for whom cognitive PVS and CS may be linked to STBs changes) using a structural equation modeling framework (e.g., mediation and moderation analyses) with more time points. These analyses present small effect sizes: however, these findings are clinically meaningful, as early identification of behaviors that increase suicide risk even slightly can prevent devastating outcomes and provide clues to better understanding STBs. One limitation of all self-report data is self-reporting biases arising from social desirability, recall period, or selective recall. It is possible that participants under-reported STBs experiences due to fears of parents and authority figures being informed of their behavior. Although, ABCD is designed to ensure youths feel comfortable providing honest answers; research assistants underwent extensive training, including motivational interviewing techniques, to alleviate concerns about disclosing responses to parents. Youths and parents were informed that only specific responses (e.g., posing an immediate and real danger to themselves or others) would be shared with parents.

## 5. Conclusions

Few studies have examined the interplay between PVS and CS in relation to ALC and STBs through an RDoC framework. Our manuscript is the first to examine PVS, CS, and STB profiles in early adolescents (ages 9–12 years) and suggests that unique profiles related to these constructs prospectively predict ALC, STBs, and their co-occurrence two years after initial assessment. A better understanding of the relationship between PVS, CS, and STBs will help to identify neurobehavioral markers of increased risk, an important first step in moving beyond clinicians’ reliance on self-report and subjective impressions to predict suicide. Further, this research can inform early intervention, precision medicine strategies, and suggest avenues for neuroscience-informed STB interventions.

## Figures and Tables

**Table 1:** Descriptive statistics of the positive valence systems (PVS) and neurocognitive systems (CS) profiles of suicidal thoughts and behaviors (STBs) at baseline enrollment (*n* = 10,414) with children (age 9 to 10 years) in the Adolescent Brain and Cognitive Development (ABCD) study.

Characteristics (*n*, %/Mean, Std)	Total Sample *N* = 10,414	Profile 1 Average PVS and CS *n* = 4940 (Reference Group)	Profile 2 High PVS and Low CS (But High Impulsivity) *n* = 1470	Profile 3 Low PVS and High CS (But Low Impulsivity) *n* = 4004	Tests for Group Differences ^†^	
					Profile 1 vs. 2 *p* Value	Profile 1 vs. 3 *p* Value
Age (years)	9.9 (0.6)	9.9 (0.6)	9.8 (0.6)	10.0 (0.6)	≤0.001	≤0.001
Sex (Biological)						
Female	4961 (47.6%)	2245 (45.4%)	568 (38.6%)	2148 (53.6%)	≤0.001	≤0.001
Male	5453 (52.4%)	2695 (54.6%)	902 (61.4%)	1856 (46.4%)		
Parental Education						
>High School Graduate	7106 (68.3%)	3210 (65%)	761 (51.8%)	3138 (78.4%)	≤0.001	≤0.001
<High School Graduate	3308 (31.7%)	1730 (35%)	709 (48.2%)	866 (21.6%)		
Ethno-Racial Identity						
White	5602 (53.8%)	2508 (50.8%)	525 (35.7%)	2569 (64.2%)	≤0.001	≤0.001
Hispanic/Latino	2086 (20.0%)	1088 (22%)	332 (22.6%)	666 (16.6%)		
Black	1422 (13.7%)	758 (15.3%)	432 (29.4%)	232 (5.8%)		
Asian/Pacific Islander	216 (2.1%)	85 (1.7%)	17 (1.2%)	114 (2.8%)		
Other	1088 (10.4%)	501 (10.1%)	164 (11.2%)	423 (10.6%)		
Lifetime Alcohol Use (ALC)						
Yes (>1 Sip of Alcohol)	985 (9.5%)	366 (7.4%)	83 (5.6%)	536 (13.4%)	≤0.001	≤0.001
Lifetime Suicidal Thoughts and Behaviors (STBs)						
Yes	739 (7.1%)	336 (6.8%)	143 (9.7%)	263 (6.6%)	≤0.001	≤0.001
Lifetime Co-Occurring ALC + STBs						
Yes	177 (1.7%)	83 (1.7%)	22 (1.5%)	71 (1.8%)	0.779	<0.001
Positive Valence Systems (PVS)						
Reward Sensitivity						
BIS Sum	9.5 (3.7)	9.4 (3.7)	9.8 (4.0)	9.6 (3.6)	0.007 **	0.278
BAS Reward Responsiveness	11.0 (2.9)	11.1 (2.9)	11.1 (3.2)	10.8 (2.8)	0.407	<0.001 ***
Cognitive Systems (CS; Neurocognition)						
Inhibitory Control/ Attention-Flanker	94.2 (8.9)	94.2 (8.4)	87.9 (11.3)	96.5 (7.4)	≤0.001 ***	≤0.001 ***
Processing Speed/ InformationProcessing–-Pattern Comparison	88.2 (14.5)	87.2 (14.1)	80.6 (14.2)	92.1 (13.8)	≤0.001 ***	≤0.001 ***
Episodic Memory-–Picture Sequence	103.1 (12.1)	100.2 (10.3)	93.2 (9.3)	110.2 (10.9)	≤0.001 ***	≤0.001 ***
Verbal Learning (RAVLT)						
Short Delay	58.8 (13.3)	55.3 (6.9)	37.9 (9.0)	70.9 (6.8)	≤0.001 ***	≤0.001 ***
Long Delay	9.2 (3.2)	8.4 (1.8)	4.3 (2.1)	12.1 (1.6)	≤0.001 ***	≤0.001 ***
Impulsivity						
Negative Urgency	8.5 (2.6)	8.5 (2.7)	9.1 (2.9)	8.3 (2.5)	≤0.001 ***	≤0.001 ***
Positive Urgency	8.0 (2.9)	8.0 (3.0)	8.9 (3.2)	7.5 (2.7)	≤0.001 ***	≤0.001 ***
Lack of Planning	7.7 (2.4)	7.7 (2.4)	8.0 (2.7)	7.7 (2.2)	≤0.001 ***	0.546
Sensation Seeking	9.8 (2.7)	9.8 (2.7)	9.6 (2.9)	9.8 (2.6)	0.014 *	0.919
Lack of Perseverance	7.0 (2.2)	7.1 (2.2)	7.6 (2.7)	6.8 (2.0)	≤0.001 ***	≤0.001 ***

Note: *p* value “*” < 0.05, “**” ≤ 0.01, “***” ≤ 0.001. ^†^ Derived from t-test for continuous variables, and chi-square tests for categorical variables with adjusted *p*-values from multiple comparisons.

**Table 2:** Fit statistics for latent profile model specification at baseline study enrollment (*N* = 10,414).

Model Specification	AIC	BIC	Entropy *	Number of Individuals per Profile						*p* Value ^†^
				1	2	3	4	5	6	
**ABCD Participants who Completed Study Enrollment at Baseline and Year 2 Follow-Up (*N* = 10,414)**										
Two-Profile	434,252	434,586	0.75	3972	6442					≤0.001
**Three-Profile**	**431,091**	**431,541**	**0.77**	**4940**	**1470**	**4004**				0.036
Four-Profile	428,304	429,823	0.739	1492	1896	3424	3602			0.085
Five-Profile	426,956	427,638	0.75	3557	763	1228	1904	2962		0.111

Note: AIC, * Entropy is an index of how well the latent profiles are separated: it ranges from zero to one with higher values to be a sign of a useful model. ^†^ Derived from the Vuong Lo Mendell Rubin adjusted test that assesses whether the number of profiles provides improved model fit compared to the model using one fewer profiles.

**Table 3:** Longitudinal multinomial logistic regression standardized models for baseline profiles (predictors) showing associations with year two follow-up classification of lifetime alcohol use (ALC), lifetime suicidal thoughts and behaviors (STBs), and lifetime co-occurring ALC + STBs (outcomes).

Profile (Predictor) (REF = Profile 1: Average PVS and CS)	Odds Ratio	95% Confidence Interval	*p* Value
Outcome: Lifetime Alcohol Use (ALC)			
Profile 2: High PVS with Low CS *(but high impulsivity)*	0.67	0.47–0.96	0.028 *
Profile 3: Low PVS with High CS *(but low impulsivity)*	1.41	1.17–1.71	≤0.001 ***
Outcome: Lifetime Suicidal Thoughts and Behaviors (STBs)			
Profile 2: High PVS with Low CS *(but high impulsivity)*	1.25	1.01–1.56	0.038 *
Profile 3: Low PVS with High CS *(but low impulsivity)*	0.95	0.80–1.13	0.593
Outcome: Lifetime Co-occurring Alcohol Use and Suicidal Thoughts and Behaviors (ALC + STBs)			
Profile 2: High PVS with Low CS *(but high impulsivity)*	0.55	0.22–1.42	0.217
Profile 3: Low PVS with High CS *(but low impulsivity)*	1.38	0.88–2.17	0.158

Note: *p*-value “*” ≤ 0.05, “***” ≤ 0.001. Covariates included in longitudinal multinomial logistic regression standardized models included age (*p* ≤ 0.001 ALC, *p* = 0.024 STB, *p* = 0.322 ALC + STBs), sex (biological; *p* = 0.001 ALC, *p* ≤ 0.001 STB, *p* = 0.002 ALC + STBs), ethno-racial identity (*p* ≤ 0.001 ALC, *p* = 0.05 STBs, *p* = 0.07 ALC + STBs), and parental education (*p* ≤ 0.001 ALC, STBs, and ALC + STBs).

## Data Availability

The ABCD data repository grows and changes over time. The ABCD data used in this report came from Annual Release 4.0, doi:10.15154/1519007. DOIs can be found at https://ndar.nih.gov/study.html?id=721) (accessed on 20 May 2022).
